# Widely Assumed but Thinly Tested: Do Employee Volunteers' Self-Reported Skill Improvements Reflect the Nature of Their Volunteering Experiences?

**DOI:** 10.3389/fpsyg.2016.00495

**Published:** 2016-04-15

**Authors:** David A. Jones

**Affiliations:** Grossman School of Business, University of VermontBurlington, VT, USA

**Keywords:** corporate social responsibility, corporate volunteerism programs, employee volunteers, employee volunteerism, community involvement, skill development, professional development, self-efficacy

## Abstract

An increasing number of companies use corporate volunteering programs (CVPs) to support and coordinate their employees' efforts to serve their communities. Among the most frequently touted benefits of such programs to sponsoring companies and employee volunteers alike is the opportunities for employees to develop tangible work-related skills through their volunteering activities. Evidence for skill development through volunteering, however, is mostly limited to the expressed beliefs of corporate leaders and employee volunteers. This study was designed to contribute to this largely anecdotal literature by testing hypotheses about the extent to which employee volunteers' self-reported skill development reflects the characteristics of the volunteers and their volunteering experiences. Study participants were 74 employee volunteers who completed a service apprenticeship managed by a U.S.-based nonprofit called Citizen Schools that partners with middle schools to extend the learning day with a combination of academic support, enrichment, and youth development activities. Data were obtained via the nonprofit's records, and surveys completed by employee volunteers before and after their service experience, including measures used to assess self-reported improvements in each of 10 work-related skills: communicating performance expectations, leadership, mentorship, motivating others, project management, providing performance feedback, public speaking and presenting, speaking clearly, teamwork, and time management. Support was found for several hypothesized effects suggesting that employees who practiced specific skills more often during their volunteering experience reported greater improvements in those skills. Improvements in some skills were higher among employee volunteers who completed a greater number of pre-volunteering preparation courses, and the effects of preparation courses were moderated by the employee volunteers' self-efficacy about improving their work-related skills on all 10 skills as hypothesized. I discuss the implications of these findings for theory and research, and provide suggestions for designing volunteer experiences that encourage service commitments from companies and their employees, and ultimately create tangible value for them and meaningful social value for their communities.

## Introduction

An increasing number of firms are developing corporate volunteering programs (CVPs) to support and coordinate their employees' efforts to serve their communities and other social and environmental causes (Peloza et al., 2009; Henning and Jones, 2013). In the U.S., for example, as the percentage of the adult population who volunteer their time each year has remained relatively stable at 25–30% (U.S. Bureau of Labor Statistics, 2014), the extent of volunteering through CVPs has grown considerably more rapidly, with over 90% of Fortune 500 companies headquartered in the U.S. having a CVP (Boccalandro, 2009). Similar growth in CVPs is observed in the U.K. and Western Europe, as well (Bussell and Forbes, 2008; Pajo and Lee, 2011).

Research shows that volunteering on one's own time outside of work is linked to employees' job performance (Rodell, 2013), and employees who volunteer through their employer's CVP report higher satisfaction and commitment (Peterson, 2004; de Gilder et al., 2005). Other studies show that CVPs are effective for attracting a greater number of job applicants, thereby increasing the likelihood of hiring high performing employees (Jones et al., 2014). Another study showed that employees who valued and appreciated their employer's CVP had stronger organizational identification and intentions to remain with their employing organization, and they performed more cooperative extra-role behaviors at work (Jones, 2010).

Of particular relevance to this study, however, is the question of whether employee volunteers can develop their work-related skills through their employer's CVP. “Opinion-poll” surveys consistently show that various parties claim that CVPs offer valuable opportunities for employees to develop tangible skills that transfer to their paid employment role (Henning and Jones, 2013). Such claims are offered by the authors of articles in the popular business press (e.g., Barbian, 2001), business leaders (e.g., Gurchiek, 2007; Lee, 2011), CVP directors (e.g., Wild, 1993), and the employee volunteers themselves (e.g., Tuffrey, 2003; Peterson, 2004). Human resources professionals likewise express this belief, with some going so far as to suggest that CVPs can effectively replace formal training and internal development programs (Points of Light Foundation, 2005).

But, as they saying goes, talk is cheap. Scholarly and practitioner-driven research in this area is almost exclusively limited to anecdotal and qualitative accounts of skill development (Geroy et al., 2000; Hall et al., 2001; Pancer et al., 2002; Graff, 2004). As the authors of several literature reviews have noted (e.g., Cihlar, 2004; Henning and Jones, 2013), the employee volunteerism literature is replete with anecdotal evidence and there is great need for more rigorous testing of theoretically-driven hypotheses, especially with respect to testing evidence for skill development through volunteering.

The present study was designed to extend and contribute to the largely anecdotal literature on skill development through employee volunteerism by testing hypotheses about the extent to which employees' self-reported skill development reflects the characteristics of the volunteers and their volunteering experiences. For instance, if skill development requires practice and employees can truly enhance their skills through volunteering, it stands to reason that employees who practice specific skills more often during their volunteering assignments will experience and report higher levels of improvements in those skills (Bartel et al., 2001). To foreshadow a second study hypothesis, individuals' self-efficacy facilitates learning and behavior change (Bandura, 1977), so if skill development truly occurs the effects of various aspects of the volunteering experiences on self-reported skill development will be stronger among employee volunteers who possess greater self-efficacy about their ability to enhance their work-related skills. With only a few notable and important exceptions (see Booth et al., 2009; Pajo and Lee, 2011; Grant, 2012), the employee volunteerism literature has not adequately considered how characteristics of the volunteering experiences affect the subsequent reactions and other outcomes among employee volunteers. As stated by Pajo and Lee (Pajo and Lee, 2011, P: 468), the nature of the literature has propagated “views of employee volunteering initiatives as relatively undifferentiated and homogenous in character,” which does not reflect the reality of these activities and, I assert, their differential effects on employee volunteers.

In the present study, I take advantage of the unique experiences that each employee volunteer encounters to test theoretically grounded hypotheses about the effects of those experiences, as well as an important personal characteristic (self-efficacy), on their self-reported improvements in 10 work-related skills. In addition to contributing to theory and offering a more rigorous approach to understanding skill development through employee volunteerism, this study has potentially important implications for promoting societal good. Should support be found for study hypotheses, it would provide what may be the strongest evidence to date for the widely-held but thinly-tested claim that employees can develop their work-related skills through volunteering. Evidence from this study might also inform future research that can provide even stronger evidence for this phenomenon, if it truly exists. The accumulation of such evidence would allow corporate policy makers to make more informed decisions and provide stronger justifications for their investments in employee volunteerism, rather than being forced to rely on anecdotal evidence and what appear to be unwarranted claims in the popular press as judged by scientific standards.

Corporate-sponsored volunteerism has been described as a *win-win-win-win-win* phenomenon, bringing the potential to provide important benefits for employee volunteers, employing organizations, volunteer organizations and community groups, individual citizens, and governments (Graff, 2004). Skill development through employee volunteerism is one of the most frequently touted reasons companies invest in CVPs (Henning and Jones, 2013), and among the top reasons employees chose to volunteer is to gain skills (Geroy et al., 2000). As such, it behooves all stakeholders involved to have a better understanding of whether the widely assumed beliefs and self-reported evidence for employee skill development through volunteering have any grounding in the nature of the associated volunteer experiences on which such claims are based.

## Prior research on skill development through company-sponsored volunteering

Evidence suggests that as many as 60% of companies that invest in a CVP do so to develop their employees' skills and competencies (Points of Light Foundation, 2000). As previously noted, numerous anecdotal and qualitative accounts show that people claim to believe that employees can develop their work-related skills through volunteering through their employer's CVP. As an illustration of such evidence from the practitioner-oriented literature, Tuffrey (2003) reported that when employee volunteers were asked what they “got out” of their involvement in a CVP, 42 and 36% endorsed the statements, “improved my team working ability” and “developed skills useful for my job,” respectively. Similar evidence can be found in the scholarly literature as well. Peterson (2004), for example, asked employees to rate items to measure their beliefs about the extent to which employees *might* develop or enhance four types of skills through participating in a CVP: teamwork, verbal, and written communication, project management, and leadership and people skills. Employees who volunteered through the CVP rated all four job skill items significantly higher than did the employees who had not volunteered through the CVP. While informative, self-reported endorsements like these provided limited evidence in support of the inference that self-reported skill improvements reflect actual enhancements in work-related skills.

Other scholars have analyzed the content of learning stories from more senior level business professionals who participated in their company's international service learning program. Pless et al.'s (2011) analysis of the “Project Ulysses” program at PricewaterhouseCoopers suggests that senior-level leaders believed they developed skills like greater cultural empathy, a broader understanding of sustainability issues, and emotional regulation. A strength of such evidence is that the self-reported learning was grounded in the executives' narratives about their international service experiences, rather than just endorsing statements about skill development. However, the nature and depth of the volunteering experiences among these senior executives are not representative of the kinds of experiences most employee volunteers have through participating in their employer's CVP. Indeed, as other researchers have observed (Wood, 2007; Pajo and Lee, 2011), most employee volunteers work in non-senior organizational roles in their paid employment context. Moreover, the improvements inferred by Pless et al. (2011) focused more on broader learning outcomes (e.g., “increases sensitivity to ethical issues”), rather than specific behavioral-based skills (e.g., time management or providing performance feedback). As such, while Pless et al. (2011) and other studies of international service assignments among senior executives offer important insights, it is questionable whether the evidence for meaningful professional development from this research generalizes to the development of specific behavior-based skills among the larger employee population who engage in community service through their employer's CVP.

In the context of the phenomenon on which the present study focuses, two studies published in high caliber scholarly journals are particularly relevant. Booth et al. (2009) obtained access to archival data from over 3600 Canadian employees, many of whom responded “yes” to questions about whether the “volunteer activities provided” them with seven different skills. Importantly, employees who spent more hours volunteering claimed they improved a significantly greater number of skills. Notwithstanding the limitations of the yes/no response format used in the archival data and the focus on broader competencies (e.g., “interpersonal skills”) rather than more specific skill areas (e.g., “teamwork skills”), in the opinion of this author these findings provide the strongest evidence to date in the published research literature because they link skill improvements to an aspect of the volunteering experience that logically relates to the development process: having more opportunity to practice work-related skills over time.

In a second particularly relevant study published in a highly reputable journal, Caligiuri et al. (2013) focused on 65 employees from a pharmaceutical company who completed volunteer assignments lasting 5.4 months, on average. Six months after returning to work, the mean response to a measure of “skill development” suggests that many believed they developed skills. However, rather than focusing on *behavior-based skills*, the measure comprised items that focused on the employee volunteers' *perspectives* back at work, (e.g., “The volunteer assignment has enabled you to bring new ideas and fresh ways of thinking or working”). The authors also measured “capability development” by averaging responses from the managers of 19 employees to two items that focused on perspectives at work. Results showed that employee-reported “skill development” was positively correlated with managerial ratings of “capacity development” (*r* = 0.35). However, this finding offers limited evidence because neither measure included items about specific skills.

Caligiuri et al. (2013) did, however, focus on skills in another measure. Employees rated how often they used eight skills while volunteering (e.g., “marketing or communications”), and responses were averaged to measure “skill utilization.” Surprisingly, skill use correlated *negatively* with self-reported “skill development” (*r* = –0.17) and managerial ratings of “capacity development” (*r* = –0.27). These unexpected findings may be due to the mismatch between the measures used to assess “skill utilization” that included behavior-based skills, versus the other measures that did not. This is unfortunate because establishing links between skill use during volunteering with self-reported skill improvements and managerial ratings of capacity development would have provided the strongest yet assessment of skill development through volunteering; indeed, to enhance professional skills, employees need opportunity to practice them, and volunteering assignments provide relatively “safe places” for doing so.

## Theory and hypotheses

The service context experienced by the participants in the present study reflected three conditions that theory and research reviewed by Caligiuri et al. (2013) suggest are important for enhancing the potential for meaningful skill development. First, the volunteering experience was *meaningful:* participants in this study completed a 10-week “apprenticeship” through which the employee volunteers drew on their professional expertise for 90 min. plus preparation time each week to teach middle school youth, discuss career opportunities, and prepare them for a public presentation of a major project. About half of the students' projects were grounded in one or more of the so-called STEM areas (science, technology, engineering, and math). Second, the experience offered *novel challenges*: managing a group hyperactive youth with little background knowledge pertaining to the subject matter being taught creates challenges that are far removed from the daily work environment of the professionals in the sample. Third, the experience was *socially supportive and interactive*: each apprenticeship was co-taught by a staff member from the nonprofit, and often among a small team of employee volunteers (among the sample used in this study, there was an average of 3.34 volunteers per apprenticeship). Thus, the service context experienced by the employee volunteers who participated in this study provided conditions that likely nurture their skill development in a general sense. Study hypotheses build on these conditions and incorporate theory about social learning and skill mastery.

Hypothesis 1 was that skill development will be higher among volunteers who have opportunities to practice a given skill more often during their volunteering experiences. Practice and repetition, unsurprisingly, are important parts of the skill mastery process (Bandura, 1997). According to one study, the accounts from graduate students who engaged in a service learning experience suggest that skill development is enhanced when volunteers have more opportunities to practice professional skills in novel and challenging contexts (Bartel et al., 2001). Moreover, the service context in the present study provided a safe and socially supportive context to practice and use skills pertaining to the meaningful and novel challenges involved, thereby creating conditions that promote skill development (Caligiuri et al., 2013).

The volunteering context in the present study was ideally suited to assessing this hypothesis about skill utilization. The service apprenticeships differed widely in the opportunities they provided for employee volunteers to use and practice each skill due to variability in the number of employee volunteers involved in each apprenticeship, the different levels of sophistication in the various project topics, and the number and characteristics of the student mentees involved (e.g., their levels of attention, aptitude and knowledge base, and motivation).

Hypothesis 1: The extent to which employees utilize each of 10 work-related skills while volunteering is associated with greater self-reported improvement in each skill.

Hypothesis 2 focused on the effects of completing pre-volunteering preparation courses on subsequent skill development (e.g., a course in *Lesson Planning*). The nonprofit agency that designed and managed the service apprenticeships offered its volunteers a total of ~5 h of support via four optional preparation courses. The preparation courses were designed to impart advice and guidance, including advice about employing different skills that the volunteers could then practice during their 10-week apprenticeship. The subsequent volunteer experiences provided the employee volunteers a relatively “safe” environment to practice and develop the work-related skills discussed in the pre-volunteering preparation courses because the employee volunteers would not face the same kinds of pressures, constraints, and consequences of failure that they might have otherwise experienced if they were to practice the same skills in their paid work settings. This socially supportive and safe environment provided through preparation courses likely enhances employee volunteers' confidence and willingness to use their skills while volunteering to achieve their meaningful objectives and overcome the novel challenges they face, which reflect the conditions that promote skill development (Caligiuri et al., 2013).

Hypothesis 2: The number of pre-volunteering preparation courses the employee volunteers complete is associated with greater self-reported improvement in each of 10 work-related skills.

Following the predictive tradition in social-cognitive and applied psychology of considering person-by situation interactions (Mischel, 1973), Hypotheses 3 and 4 focused on a characteristic of the individual employee volunteer that moderates the strength of the situation-based effects specified in Hypotheses 1 and 2: Self-efficacy, which refers to a person's confidence and belief about having the capacity to execute behaviors that ultimately achieve a desired performance level in a specific domain (Bandura, 1977).

Self-efficacy is well recognized as an important factor in learning and development, behavioral change, and the achievement of specific goals and performance objectives (Stajkovic and Luthans, 1998; Bandura, 2001). Self-efficacy contributes independently to subsequent performance after controlling for ability and prior performance levels because individuals with higher self-efficacy put forth greater effort to learn a new skill or change a behavioral pattern, and they are apt to sustain that effort in the face challenges, difficulties, and adversity (Bandura, 1997). Through the persistence enhancing effects of self-efficacy, individuals can work toward skill improvement and mastery even in the presence of psychologically threatening or uncomfortable contexts (Bandura, 2001), such as the unfamiliar contexts in which employee volunteers often operate. Employee volunteers with higher self-efficacy will be more persistent in using and developing their skills to achieve their objectives even in the face of challenges they might encounter in the novel and unfamiliar settings in which they volunteer that reflect the kinds of novel and meaningful challenges that are believed to foster skill development (Caligiuri et al., 2013). Accordingly, I hypothesized that the effects of skill use and the number of completed preparation courses on skill development are stronger among employee volunteers with higher self-efficacy about their ability to improve their work-related skills.

Hypothesis 3: Pre-volunteering self-efficacy about the ability to improve work-related skills moderates the effects of skill utilization on skill improvement for each of 10 work-related skills, such that the relationships are stronger when the employee volunteers' prior self-efficacy is higher.

Hypothesis 4: Pre-volunteering self-efficacy about the ability to improve work-related skills moderates the effects of the number of pre-volunteering preparation courses completed on skill improvement for each of 10 work-related skills, such that the relationships are stronger when the employee volunteers' prior self-efficacy is higher.

## Materials and methods

### Volunteering context

Participants were employees who, with the encouragement and support of their employers, completed a 10 week apprenticeship as volunteer Citizen Teachers through the U.S.-based nonprofit called Citizen Schools. Citizen Schools is a national nonprofit that partners with middle schools to extend the learning day with a combination of academic support, enrichment, and youth development activities.

Citizen Schools coordinates and manages “apprenticeships,” a project-based course and mentorship model led by community volunteers called “Citizen Teachers,” many of whom are recruited from the people employed by a set of committed corporate partners. Citizen Teachers may elect to teach apprenticeship classes on various topics including financial planning, law and blogging; nearly half of the Citizen Teachers chose to cover topics grounded in the science, technology, engineering, and math disciplines. Citizen Teachers, who volunteer individually or in small groups, meet with students for 90 min. once per week to teach them about selected topics and career opportunities and to prepare them for a public presentation of their projects at the end of the 10-week apprenticeship. Each apprenticeship is co-taught by a member of Citizen Schools' staff. Because apprenticeships take place during typical business hours, Citizen Schools and its volunteers rely on support from the volunteers' employing organizations.

In addition to lecture preparation time and 90 min. in class each week, at the time of the study the Citizen Teachers were offered four optional pre-volunteering preparation courses comprising ~5 h in total. For instance, two particularly important preparation courses were on *Lesson Planning* and *Apprenticeship Design*. Thus, the employee volunteers invested about 20–35 total hours of volunteer work throughout their apprenticeship experience.

### Study participants

Participants were 74 employee volunteers, with each gender represented relatively equally (38 females and 36 males). The employee volunteers were encouraged and supported by their employers (Cognizant Technology Solutions Inc., Google, Fidelity Investments, and Cisco Systems) to complete the 10-week apprenticeship in fall 2012 or spring 2013. The volunteers worked in seven U.S. states: California (*n* = 8), Illinois (*n* = 9), Massachusetts (*n* = 15), New Jersey (*n* = 11), New Mexico (*n* = 2), New York (*n* = 21), and North Carolina (*n* = 8).

The volunteers averaged 34 years of age (ranging from 22 to 63 years) and about 4 years of tenure in their employing organization, ranging from as little as 1 month to over 15 years of tenure with their employer at the time of the pre-volunteering survey used in this study. Their highest levels of education obtained included a technical diploma or other training (*n* = 3), an undergraduate degree for half of the study participants (*n* = 37), a Master's degree (*n* = 28), or a doctorate/Ph.D. (*n* = 6). Their average amount of lifetime work experience was 12 years and 11 months, ranging from 9 months to 47 years. Based on the job functions the employee volunteers listed, a conservative estimate is that about one-third of the CTs (*n* = 24, 32%) performed work pertaining to the STEM areas, although the true percentage is likely higher because not included in this estimate were CTs working in Consulting, Business Unit Management, and other functions that plausibly pertain to the STEM areas given the nature of the employing organizations involved.

### Study procedure and measures

Data used for hypothesis testing included the number of pre-volunteering preparation courses completed by each employee volunteer obtained with their consent from records provided by Citizen Schools. The employee volunteers completed online surveys before the start of their apprenticeship experience, and 6–8 weeks after its end. Both surveys mostly comprised measures used for organizational development and purposes that were of interest to Citizen Schools and the participating employers and unrelated to the present study. The pre-apprenticeship survey included demographic, volunteering, and work history questions, and embedded among other measures was a single item measure of self-efficacy about skill improvement, which was responded to on a scale from 1 (*Strongly Disagree*) to 7 (*Strongly Agree*). Reflecting defining features of the self-efficacy construct and recommendations about its measurement (Bandura, 2006), the self-efficacy item constructed for this study focused on the respondent's confidence about a context-specific ability: “I am confident about my ability to develop and improve my work-related skills.”

The post-apprenticeship survey included an open-ended question about whether and how the employee volunteers believed they benefitted from their volunteering experience, without including references to skill development or any other potential benefits. After completing other measures not pertinent to the present study, respondents completed 10 items used to measure skill improvement in each of 10 work-related skills. The employee volunteers were asked to compare their current levels of each skill to their prior levels of that skill during a specified month and year, which corresponded to the period immediately before they had started their apprenticeship experience. Each skill improvement item began with, “Compared to [*month/year*], my skills at [*one of 10 work-related skills*] are…,” and the response options allowed for the possibility of skill declines as well as improvements, ranging from one to five (*Weaker, About the Same, A Little Stronger, Stronger*, and *Much Stronger*). The 10 work-related skills measured in this study were: “communicating performance expectations,” “leadership,” “mentorship,” “motivating others,” “project management,” “providing performance feedback,” “public speaking and presenting,” “speaking clearly,” “teamwork,” and “time management.”

Decisions about the selection and wording of the 10 skills were made through the following process. I first created an initial list of skills based on three considerations: their relevance to the apprenticeship service experience based on materials provided by Citizen Schools, their relevance to most professional employees' paid work contexts, and their grounding in skills assessed in prior research on this topic. Pertaining to the latter, I adapted the wording used in prior items to focus on more specific skills in this study relative to the more general skills measured by other researchers. For example, grounded in Booth et al.'s (2009) measure of “communication skills,” I created items to measure “communicating performance expectations,” “providing performance feedback,” “public speaking and presenting,” and “speaking clearly.” I then discussed this initial list of skills with subject matter experts from Citizen Schools, including former Citizen Teachers, and refined the list accordingly. Representatives from each corporate partner reviewed all survey items and had opportunity to opine about the relevance of the 10 skills to their employees' paid work contexts, and they did not suggest any wording changes or item removals pertaining to the 10 skills.

After completing the 10 skill improvement items, the employee volunteers were asked to respond to items used to measure skill utilization. Respondents rated how often they used each of the 10 work-related skills during their volunteering experience on a scale from 1 (*Never*) to 7 (*Every Day*).

## Results

### Self-reported skill improvements

When asked to respond to an open-ended question about the potential benefits they received from their volunteering experience, 32% of the employees wrote comments pertaining to skill development (e.g., “*It improved my public speaking skills*,” “*Improving my leadership*,” “*How to better manage a project with peers*,” and “*I benefited by improving my leadership and organizational skills”*) or the opportunity to practice or gain confidence in their skills (e.g., “*The experience challenged my communication skills in ways I am not challenged at work,” Developed my confidence, leadership, and presentation skills,”* and “*Professional growth; Public speaking; More confidence in my abilities”*).

Responses to the self-reported skill improvement items showed that compared to before they started their service apprenticeships, about 40–45% of the employee volunteers claimed some level of improvement in skills pertaining to leadership, mentorship, motivating others, project management, and public speaking and presenting. About 30–35% claimed improvements in skills pertaining to communicating performance expectations, providing performance feedback, speaking clearly, teamwork, and time management.

### Hypothesis testing

I assessed empirical justification for including nine demographic, volunteering, and work history variables as potential control variables in the models. Regression analyses showed that self-reported improvements on each of the 10 skills did not systematically differ as a function of any of six variables for which there was complete data across the sample: gender, age, education level, lifetime work experience, employment tenure, and the number of service apprenticeships they had completed prior to the one in which they were most recently engaged. Specifically, across the 60 associated coefficients, only one was significant: employment tenure had a small and marginally significant effect on motivating others (*b* = –0.01, *p* = 0.051). Moreover, self-reported skill improvements did not systematically differ based on three other variables for which there were missing data: the employee volunteers' receipt of other job-related training during the period in which study data were collected, whether they managed or supervised others, or the length of time they had managed others. Accordingly, none of these demographic, volunteering, and work history variables were used as control variables in hypothesis testing.

Table 1 shows the means, standard deviations, and correlations among study variables, and Tables 2, 3 display the results from the regression models used for hypothesis testing. Each self-reported skill development variable was regressed on skill utilization (i.e., the use of that skill during the volunteering experience) and the number of pre-volunteering preparation courses entered in Step 1, self-efficacy about skill improvement in Step 2, and the two moderator terms in Step 3. I assessed all hypotheses using two-tailed tests and the normative 0.05 alpha level.

**Table 1:** **Means, standard deviations, and correlations among study variables**.

**Variable**	***M***	***SD***	**1**	**2**	**3**	**4**	**5**	**6**	**7**	**8**	**9**	**10**	**11**	**12**	**13**	**14**	**15**	**16**	**17**	**18**	**19**	**20**	**21**
1. SI communicating perf. expectations	2.50	0.85																					
2. SI leadership	2.65	0.93	0.52																				
3. SI mentorship	2.86	1.03	0.50	0.74																			
4. SI motivating others	2.70	0.96	0.56	0.73	0.74																		
5. SI project management	2.69	0.99	0.73	0.67	0.62	0.61																	
6. SI providing performance feedback	2.49	0.83	0.78	0.67	0.64	0.60	0.68																
7. SI public speaking and presenting	2.80	0.98	0.69	0.72	0.70	0.74	0.64	0.53															
8. SI speaking clearly	2.53	0.86	0.78	0.64	0.62	0.69	0.67	0.78	0.68														
9. SI teamwork	2.51	0.91	0.69	0.75	0.65	0.74	0.74	0.77	0.70	0.66													
10. SI time management	2.55	0.89	0.73	0.62	0.59	0.53	0.72	0.78	0.58	0.65	0.76												
11. SU communicating perf. expectations	4.87	1.81	0.27	0.22	0.20	0.37	0.15	0.30	0.28	0.29	0.30	0.18											
12. SU leadership	5.42	1.63	0.27	0.18	0.23	0.32	0.16	0.24	0.24	0.23	0.26	0.17	0.58										
13. SU mentorship	5.39	1.88	0.17	0.10	0.18	0.17	0.01	0.14	0.20	0.20	0.09	0.07	0.39	0.52									
14. SU motivating others	5.77	1.59	0.20	0.09	0.21	0.21	0.06	0.17	0.19	0.11	0.16	0.08	0.53	0.71	0.72								
15. SU project management	5.07	1.93	0.28	0.10	0.25	0.25	0.26	0.28	0.22	0.17	0.25	0.19	0.36	0.52	0.52	0.46							
16. SU providing performance feedback	4.49	1.91	0.33	0.17	0.19	0.30	0.13	0.34	0.23	0.29	0.29	0.20	0.81	0.48	0.42	0.49	0.34						
17. SU public speaking and presenting	5.48	1.77	0.22	0.10	0.18	0.23	0.13	0.18	0.28	0.18	0.18	0.17	0.66	0.63	0.50	0.68	0.41	0.54					
18. SU speaking clearly	5.87	1.57	0.17	0.06	0.17	0.24	0.07	0.10	0.16	0.10	0.13	0.06	0.54	0.64	0.41	0.62	0.34	0.51	0.79				
19. SU teamwork	6.06	1.41	0.22	0.10	0.16	0.15	0.15	0.25	0.10	0.16	0.18	0.17	0.31	0.69	0.41	0.59	0.51	0.28	0.49	0.53			
20. SU time management	5.63	1.74	0.20	0.13	0.21	0.31	0.17	0.22	0.12	0.22	0.20	0.18	0.49	0.75	0.44	0.56	0.56	0.47	0.55	0.51	0.51		
21. Pre-volunteering preparation courses	1.57	1.60	0.29	0.13	0.26	0.16	0.21	0.26	0.30	0.31	0.13	0.26	0.08	0.08	0.10	0.07	0.12	0.06	0.21	0.07	0.04	0.07	
22. Self-efficacy about skill improvement	6.35	0.73	0.20	0.08	0.12	0.13	0.00	0.21	0.06	0.22	0.08	0.06	0.05	0.22	0.07	0.09	0.07	0.11	0.01	0.09	0.12	0.15	0.18

*N = 74. SI, Skill Improvement. SU, Skill Utilization (during volunteering). Perf., Performance. Correlations are significant between 0.23 and 0.30 at p < 0.05, between 0.31 and 0.39 at p < 0.01, and exceeding 0.40 at p < 0.001*.

**Table 2:** **Regression results predicting employee volunteers' self-reported skill improvements in communicating performance expectations, leadership, mentorship, motivating others, and project management**.

**Regression step predictors**	**Self-reported skill improvements**									
	**Communicating performance expectations**		**Leadership**		**Mentorship**		**Motivating others**		**Project management**	
	***b***	**SE**	***b***	**SE**	***b***	**SE**	***b***	**SE**	***b***	**SE**
Step 1:	*R^2^* = 0.15^**^		*R^2^* = 0.05		*R^2^* = 0.09^*^		*R^2^* = 0.07^†^		*R^2^* = 0.10^*^	
Skill utilization	0.12^*^	0.05	0.10	0.07	0.09	0.06	0.12^†^	0.07	0.12^*^	0.06
Preparation courses	0.15^*^	0.06	0.07	0.07	0.16^*^	0.07	0.09	0.07	0.11	0.07
Step 2:	Δ*R^2^* = 0.02		Δ*R^2^* = 0.00		Δ*R^2^* = 0.00		Δ*R^2^* = 0.01		Δ*R^2^* = 0.00	
S.-efficacy	0.16	0.13	0.04	0.15	0.09	0.16	0.12	0.16	–0.07	0.16
Step 3:	Δ*R^2^* = 0.07		Δ*R^2^* = 0.11^*^		Δ*R^2^* = 0.08^*^		Δ*R^2^* = 0.10^*^		Δ*R^2^* = 0.10^*^	
S.-efficacy × skill utilization	0.03	0.07	–0.06	0.07	–0.12	0.09	–0.03	0.08	–0.12	0.08
S.-efficacy × prep. courses	0.19^*^	0.08	0.27^**^	0.09	0.28^*^	0.11	0.29^**^	0.10	0.26^**^	0.10
Total Model *R^2^*	0.24^**^		0.16^*^		0.18^*^		0.18^*^		0.20^**^	

N = 74. S.-efficacy, Self-Efficacy about Skill Improvement.

† p < 0.10.

* p < 0.05.

** *p < 0.01*.

**Table 3:** **Regression results predicting employee volunteers' self-reported skill improvements in providing performance feedback, public speaking and presenting, speaking clearly, teamwork, and time management**.

**Regression step predictors**	**Self-reported skill improvements**									
	**Providing performance feedback**		**Public speaking and presenting**		**Speaking clearly**		**Teamwork**		**Time management**	
	***b***	**SE**	***b***	**SE**	***b***	**SE**	***b***	**SE**	***b***	**SE**
Step 1:	Δ*R^2^* = 0.17^**^		Δ*R^2^* = 0.14^**^		Δ*R^2^* = 0.10^*^		Δ*R^2^* = 0.05		Δ*R^2^* = 0.09^*^	
Skill utilization	0.14^**^	0.05	0.13^*^	0.06	0.04	0.06	0.11	0.08	0.09	0.06
preparation courses	0.13^*^	0.06	0.16^*^	0.07	0.16^**^	0.06	0.07	0.07	0.14^*^	0.06
Step 2:	Δ*R^2^* = 0.02		Δ*R^2^* = 0.00		Δ*R^2^* = 0.03		Δ*R^2^* = 0.00		Δ*R^2^* = 0.00	
S.-efficacy	0.16	0.13	0.02	0.15	0.20	0.14	0.04	0.15	−0.02	0.14
Step 3:	Δ*R^2^* = 0.06^†^		Δ*R^2^* = 0.12^**^		Δ*R^2^* = 0.18^***^		Δ*R^2^* = 0.10^*^		Δ*R^2^* = 0.09^*^	
S.-efficacy × skill utilization	−0.01	0.06	−0.05	0.07	−0.08	0.07	−0.04	0.09	−0.03	0.06
S.-efficacy × prep. courses	0.18^*^	0.08	0.32^**^	0.10	0.35^***^	0.09	0.26^**^	0.09	0.23^**^	0.09
Total Model *R^2^*	0.25^**^		0.26^**^		0.31^***^		0.15^*^		0.18^*^	

N = 74. S.-efficacy, Self-Efficacy about Skill Improvement.

† p < 0.10.

* p < 0.05.

** p < 0.01.

*** *p < 0.001*.

Hypothesis 1 was that skill utilization during volunteering is positively associated with skill improvement. Tables 2, 3 show that support for this hypothesis was found through the significant effects from Step 1 on improvements in four skills: communicating performance expectations, project management, providing performance feedback, and public speaking and presenting. On the other six skills, support was not found for Hypothesis 1 (leadership, mentorship, motivating others, speaking clearly, teamwork, and time management), although a marginally significant trend was found on motivating others (*p* < 0.10).

Hypothesis 2 was about the effects of the number of pre-volunteering preparation courses completed on improvement in each skill. As seen in Tables 2, 3, support was found through significant effects reported in Step 1 on six skills: communicating performance expectations, mentorship, providing performance feedback, public speaking and presenting, speaking clearly, and time management. Hypothesis 2 was not supported for the other four skills (leadership, motivating others, project management, and teamwork). Across the 20 coefficients tested to assess Hypotheses 1 and 2, all relationships were positive as expected.

Hypotheses 3 and 4 were that pre-volunteering levels of self-efficacy about skill improvement moderates the effects of skill utilization and preparation courses. Results reported for Step 3 in Tables 2, 3 show that after accounting for the effects of skill utilization, preparation courses, and self-efficacy, none of the moderator terms representing the interaction between self-efficacy and skill utilization were significant. Therefore, no support was found for Hypothesis 3. Pertaining to Hypothesis 4, however, the moderator terms comprising the number of preparation courses completed and self-efficacy were significant in predicting self-reported improvements in all 10 skills.

To understand the nature of these significant interactions, I tested simple slopes by regressing self-reported improvement in each skill on the number of courses completed within higher and lower groups on self-efficacy about skill improvement. In the context of the modest sample size and distribution of responses to the self-efficacy measure, I was unable to create groups using a ±1 *SD* approach, for example. I instead used the distribution of scores to identify the point at which two similarly sized groups could be created while comprising enough respondents to allow for meaningful inferences based on simple slope tests. I created a higher self-efficacy group comprising individuals who responded with a seven to indicate their “strong agreement” with the item (*n* = 35), and a lower self-efficacy group comprising all other individuals in the sample (*n* = 39). Table 4 displays the results of these simple slope tests: Across all 10 skill improvement variables, the simple slope for the effect of preparation courses was not significant among the lower self-efficacy group, and the slope was significant and positive among the higher self-efficacy group. Thus, Hypothesis 4 was fully supported.

**Table 4:** **Tests of simple slopes within lower and higher groups on self-efficacy about skill improvement: effects of the number of pre-volunteering preparation courses completed on self-reported improvements in 10 work-related skills**.

**Self-reported skill improvement**	**Lower self-efficacy about skill improvement (*****n*** = **39)**			**Higher self-efficacy about skill improvement (*****n*** = **35)**		
	**Effects of preparation courses**			**Effects of preparation courses**		
	***b***	***SE***	***R*^2^**	***b***	***SE***	***R*^2^**
Communicating performance expectations	−0.01	0.07	0.00	0.30^**^	0.09	0.25
Leadership	−0.17	0.09	0.10	0.35^**^	0.09	0.30
Mentorship	−0.03	0.10	0.00	0.38^**^	0.10	0.29
Motivating others	−0.12	0.09	0.04	0.32^**^	0.10	0.24
Project management	−0.06	0.10	0.01	0.34^**^	0.10	0.28
Providing performance feedback	−0.04	0.07	0.01	0.31^**^	0.09	0.27
Public speaking and presenting	−0.04	0.09	0.01	0.44^***^	0.10	0.38
Speaking clearly	−0.08	0.07	0.04	0.40^***^	0.09	0.39
Teamwork	−0.13	0.08	0.06	0.29^**^	0.10	0.21
Time management	−0.03	0.09	0.00	0.33^**^	0.09	0.29

** p < 0.01.

*** *p < 0.001*.

### *Post-hoc* analyses

I conducted post hoc analyses using measures of skill utilization and skill improvement that were aggregated across the items pertaining to all 10 skills. Both aggregate variables are formative measures comprising 10 indicators that define and cause the measured construct, rather than reflective measures for which the indicators reflect an underlying latent construct. The indicators of a formative measure are not interchangeable with other indicators, nor do the indicators necessarily covary given their potentially different antecedents and consequences (Podsakoff et al., 2006). In the case of these two formative measures, however, the indicators did covary to a considerable degree, as suggested by the Cronbach alpha internal consistency estimates of 0.92 and 0.95 for the aggregated skill utilization and skill improvement measures, respectively.

I regressed the aggregate measure of skill improvement on the aggregate measure of skill utilization and other study variables entered in the regression model using the same approach described for hypothesis testing. The model's total *R*^2^ value of 0.29 was statistically significant (*p* < 0.001). Consistent with the significant effects that provided support for Hypotheses 1 and 2, results from Step 1 showed that the aggregate measure of skill improvement was significantly predicted by the aggregate skill utilization measure (*b* = 0.16, *SE* = 0.07, *p* < 0.05) and the number of pre-volunteering preparation classes completed (*b* = 0.12, *SE* = 0.05, *p* < 0.05). Consistent with the absence of support found for Hypothesis 3 and the full support found for Hypothesis 4, results from Step 3 showed that self-efficacy about skill improvement did not interact with the aggregate skill utilization variable (*b* = –0.04, *SE* = 0.07, *p* > 0.05), but it did significantly interact with the number of preparation courses completed in predicting the aggregate measure of skill improvement (*b* = 0.26, *SE* = 0.07, *p* < 0.05). The same pattern of simple slopes was observed as the pattern reported for the tests of Hypothesis 4: the effect of the number of preparation courses completed was not significant among the lower self-efficacy group (*b* = –0.07, *SE* = 0.07, *p* > 0.05), but it was significant among the higher self-efficacy group (*b* = 0.35, SE = 0.08, *p* < 0.001), explaining 37% of the variance in the aggregated measure of skill improvement.

## Discussion

An increasing number of companies invest in corporate volunteering programs to coordinate and foster their employees' volunteer activities and community service, and anecdotal evidence shows that multiple parties believe that employee volunteers can develop work-related skills through volunteering (Henning and Jones, 2013). Study results showed that, in response to an open-ended question about the potential benefits they received from volunteering, approximately one-third of the employee volunteers mentioned skill development (e.g., “*It improved my public speaking skills*,” “*Improving my leadership*”). Responses to survey items showed that about one-third to almost one-half of the employee volunteers claimed some level of skill improvement in each of the 10 work-related skills measured in this study: communicating performance expectations, leadership, mentorship, motivating others, project management, providing performance feedback, public speaking and presenting, speaking clearly, teamwork, and time management.

While the above findings are intriguing, as I stated at the outset of this article, “talk is cheap.” Open-ended responses and the distributions of self-reported responses to statements about skill improvement offer the same kinds of anecdotal evidence that pervades the scholarly and practitioner literatures on this topic that the authors of literature reviews have lamented (Cihlar, 2004; Henning and Jones, 2013). Far more important for the purpose of advancing this literature are study findings from the tests of hypotheses that provide answers to the following question: Do self-reported skill improvements actually reflect the characteristics of the employee volunteers and the nature of their volunteering experiences, as theory and common sense dictate they should if employee volunteers are truly developing their work-related skills through volunteering?

Tests of study hypotheses showed that the employee volunteers who had more opportunities to practice four work-related skills while volunteering reported significantly greater improvements in those same four skills. If skill development truly occurs through volunteering, such findings are not surprising given the similar effects reported among students in service learning contexts (Bartel et al., 2001), theory about skill mastery (Bandura, 1997), and common sense. Other results showed that self-reported improvements in six skills were significantly higher among employee volunteers who completed a greater number of pre-volunteering preparation courses. Moreover, and as I soon discuss in the context of well-established theory (Bandura, 1977, 2001; Locke and Latham, 2002), the effects of these preparation courses were moderated by the employee volunteers' self-efficacy about improving their work-related skills, such that the preparation courses were associated with greater improvements in all 10 skills among employee volunteers with higher self-efficacy. In the sections that follow I discuss the implications of these findings for theory and research, review study limitations, and provide practical suggestions for designing and managing volunteer experiences to create greater value for all stakeholders involved.

### Implications for theory and research

This study contributes to the scholarly literature by moving beyond findings about what employees claim to believe about skill development through employee volunteering (Peterson, 2004). While this study certainly has its own limitations that I later describe, it addresses limitations described earlier with respect to what are perhaps the two strongest published studies in this area (Booth et al., 2009; Caligiuri et al., 2013), such as by demonstrating that ratings of the extent of employee volunteers' use of specific skills during a volunteering assignment predicts the extent of their self-reported improvements in those same skills. In addition to reasons just described, I expected to find such effects because the volunteering context in this study conformed to three conditions that Caligiuri et al. (2013) described as being critically important for meaningful skill development through volunteering: the volunteering experiences were *meaningful*, offered *novel challenges*, and were *socially supportive and interactive*. In future studies, researchers should measure and test these and other characteristics of the volunteering experience to help fill the gap highlighted by Pajo and Lee (2011) in the scholarly understanding of how such characteristics affect subsequent outcomes among employee volunteers.

Other study results inform theory and future research in novel ways. Findings showed that the completion of a greater number of pre-volunteering preparation courses predicted improvements in all 10 skills among employee volunteers who had higher self-efficacy about skill improvement, but not among those with lower self-efficacy. Research on goal setting provides insight into the potential reasons the hypothesized interactive patterns were consistently found for the effects of preparation courses, despite the use of severely underpowered tests that created considerable challenges to detecting any significant interactions that truly exist. Research on goal setting shows that individuals who have higher self-efficacy set more challenging goals, develop better task strategies to attain them, and are less likely to become demoralized in the face of setbacks (Locke and Latham, 2002). Faced with complex challenges, people with higher levels of self-efficacy are more likely to develop task relevant strategies that, in turn, enhance performance (Winters and Latham, 1996; Seijts and Latham, 2001). As such, during their participation in pre-volunteering preparation courses, the employee volunteers with higher self-efficacy about skill improvement were apt to formulate strategies to use, improve, and apply their skills to create a rewarding apprenticeship experiences for both themselves and the youth they mentor, hence leading to greater skill development over the course of their 10-week apprenticeship. Future research on skill development through volunteering should explore the strategies volunteers develop to achieve challenging objectives and examine the extent to which those strategies involve the use and honing of specific work-related skills.

### Study limitations and other directions for future research

The inferences that can be drawn from these results are tempered by a number of limitations of the study design. Among the most important limitations is that the measures of skill development are subject to the usual pitfalls of relying on self-reported data that may be heightened in the present context. The employee volunteers may have been motived to exaggerate their claims about skill improvement in an attempt to benefit the nonprofit agency involved or to justify to themselves or their employer that their time volunteering was time well spent. For this reason, I emphasized through the survey instructions the importance of honest responding, that only the researcher would have access to their survey responses, and that no individual responses would be shared with employers or any other party without maintaining confidentiality and anonymity. While this study extends prior research by linking self-reported skill development to characteristics of employee volunteers and their volunteer experiences, evidence of skill development through measures obtained via supervisor report or some other independent source would provide significantly stronger evidence than the present study can offer.

While measuring improvement through self-report is a meaningful limitation, there are good reasons to reject common method variance as a viable explanation for the majority of significant study results. Exactly 80% of the significant effects found in this study that provided support for study hypotheses utilized a variable that was not self-reported and was instead obtained from Citizen Schools' records: the number of pre-volunteering preparation courses completed. Specifically, this variable was involved in 16 of the 20 significant coefficients that provided support for study hypotheses as reported in Tables 2, 3, including all 10 significant interaction effects. The other variable involved in the 10 significant interaction effects (self-efficacy about skill improvement) was measured 16–18 weeks before the self-reported measures of skill improvement, and temporal separation between predictors and criteria is a recommended method to reduce common method variance (Podsakoff et al., 2003). Moreover, the tests of simple slopes conformed precisely to the theorized and hypothesized interactive patterns across all 10 skill improvement variables, which provides convincing evidence that common method bias is not driving this consistent pattern of effects (Siemsen et al., 2010). Accordingly, I can confidently rule out common method variance as a meaningful threat to the inferences drawn from most of the significant effects reported in this study.

Another design limitation is the use of single-items to measure most study variables. Single-item scales are often criticized for being potentially unreliable indicators of the underlying construct, and for insufficiently capturing a given construct space. Notwithstanding these potential issues, evidence suggests that their use is not always as problematic as some people assume. Wanous et al. (1997) provided meta-analytic evidence supporting the validity of single-item measures of job satisfaction, reporting a corrected mean correlation of 0.67 between single- and multiple-item measures, as well as test-retest reliabilities for single-item scales for which the lowest observed value was 0.70. Nagy (2002) likewise found evidence supporting the use of single-item measures of satisfaction facets, which correlated significantly with multiple-item measures of the same facets as assessed by the Job Descriptive Index (values ranged between 0.60 and 0.72), and the single-item measures explained similar amounts of variance in self-reported job performance and turnover intentions relative to the multiple-item versions. Similar findings and other types of validity evidence that support the use of single-item measures have been reported for measures of global self-esteem (Robins et al., 2001), burnout (Rohland et al., 2004), and stress symptoms (Elo et al., 2003). Of particular relevance is a study conducted over the course of six months involving over 300 inpatients at risk for substance use relapse (Hoeppner et al., 2011). Analyses of a single-item measure of self-efficacy provided consistent evidence for its convergent and discriminant validity with a 20-item self-efficacy measure and a temptation sub-scale, respectively. Moreover, this study reported that the single-item self-efficacy measure predicted relapse at 1, 3, and 6 month post-discharge, whereas the 20-item measure of self-efficacy did not.

Perhaps the largest limitation of this study is that the self-reported skill improvements among the employee volunteers were not compared to the same self-reported ratings among a comparable control group of employees who did not volunteer. As such, I am unable to estimate or rule out the potential influence of history and maturation effects on these findings (e.g., the possibility that skill improvements were due to common experiences at work during the time of the study such as an annual performance review meeting, or the natural skill development that occurs over time regardless of volunteering experiences). Results did show that skill development was unrelated to a single item used to assess the extent of any other work-related training they received during the apprenticeship period, but unknown is whether and the extent to which non-volunteers believe they develop their work-related skills to comparable degrees during the same period of time. A pre-post volunteering treatment-control group design would offer an especially strong assessment of skill development among employees who volunteered, relative to employees who did not. Specifically, researchers should measure the levels of each skill in both the treatment and control groups before and after individuals in the treatment group complete their volunteering experience to provide a rigorous assessment of skill development via volunteering relative to any potential skill changes among employees in the control group.

Also unknown is whether and the extent to which any skill development occurred *during* one or more of the four pre-volunteering preparation courses versus occurring *subsequent* to those courses as a result of their influence on shaping the employee volunteers' use of specific work-related skills during later volunteering experiences. Based on information provided by the nonprofit about the preparation courses, the evidence for interactive effects with self-efficacy, and the goal setting theory and research I described previously (Locke and Latham, 2002), I suspect that most skill development occurred *subsequent* to the courses and that these employee volunteers likely developed their apprenticeship strategies *during* these preparation courses, but additional research would be needed to tease apart these potential effects. Notably, evidence for skill development from this study is not limited to the findings involving the completion of these courses, as the significant effects found for skill utilization in predicting self-reported improvements in four work-related skills were found after controlling for the effects of the preparation courses that were tested in the same step of the regression models.

### Suggestions for practice and volunteerism program design

These limitations notwithstanding, the results of this study provide insights about designing and managing CVPs for the most immediate and direct impact on skill development, and indirect impact on employer and community well-being. Below I provide suggestions for designing and managing CVPs and the experiences to encourage service commitments from companies and their employees, and ultimately create tangible value for them and meaningful social value in their communities. I stress, however, that the merits of these suggestions are predicated on the extent to which the findings from this study reflect true improvements in work-related skills among these (and other) employee volunteers, which is a question that can only be definitely resolved through more research conducted with higher levels of scientific rigor than extant research to date, including that of the present study. The following suggestions for practice are offered against the backdrop of this important caveat, which I refrain from repeating with each suggestion.

Through many CVPs, companies allocate 25 to 40 h of paid volunteering hours each year to their full time employees (Henning and Jones, 2013). The evidence for skill development from this study is based on a similar number of volunteer hours: each service apprenticeship involved ~20–35 h, including the 15 h of in-class volunteering, preparation time, and time spent in the optional pre-volunteering preparation courses. This evidence for employees' skill development through volunteering adds to the growing business case for the multiple pay-offs employers reap from their investments in CVPs, including but not limited to evidence showing that such programs enhance corporate reputation and brand equity (Cihlar, 2004; Peloza et al., 2009; Henning and Jones, 2013); help firms attract more job applicants, which increases selection system utility (Jones and Willness, 2013; Jones et al., 2014); and improve employee retention, commitment, and extra-role cooperative behaviors at work (Peterson, 2004; de Gilder et al., 2005; Jones, 2010). While I am unaware of any rigorous assessments of cost-to-benefit ratios or the opportunity costs involved in creating and maintaining different types of CVPs, I am an eager consumer of the research evidence and am as convinced as a great many corporate leaders appear to be about the meaningful returns from investments in CVPs. The extant evidence justifies a focus of efforts not on understanding *whether* CVPs payoff for the sponsoring organizations, but *how* CVPs can best be managed to create meaningful shared value for companies, employee volunteers, and the causes and communities they serve.

The results of this study suggest that to enhance skill development CVPs should be designed and managed to provide employee volunteers with greater opportunities to practice and hone their work-related skills. Such arrangements may be best achieved through alliances between employers and the nonprofits that are served by employee volunteers (Booth et al., 2009). Presumably, opportunities to practice skills should occur through service activities that are meaningful and represent novel challenges while being socially supportive and interactive (Caligiuri et al., 2013). Other findings suggest that these types of volunteer activities should be targeted toward the broader employee population, rather than, overtly focusing on managing a firm's millennial-generation employees.

The sample included employee volunteers that ranged in age between 22 and 63 years, and age variability was unrelated to the levels of self-reported skill improvement across all ten skills measured in this study. This findings may be surprising and disappointing to corporate leaders and HR professionals who view CVPs as an important element of their strategies to attract and recruit millennial-generation employees, and of relevance to this study, to enhance their talents. The emphasis on using CVPs and related practices for managing “generation Y” employees is present in the popular business press (e.g., Epstein and Howes, 2006; Gurchiek, 2007), and has been a focus among the representatives of almost every company this author has worked with to develop or assess CVPs. But many age-related stereotypes in organizational and workplace contexts simply do not fit the data (e.g., Ng and Feldman, 2008, 2012), much like stereotypes about environmental behaviors among younger versus older-aged employees (Wiernik et al., 2013).

Generation Y-focused corporate leaders and HR professionals who interpret the lack of age-related effects in this study as something other than “good news” should instead view the lack of such effects as “great news.” The results of this study suggest that skill development through volunteering is not limited to younger workers, or to those with less job experience, or whether their highest degree held was a technical diploma or a doctorate, among other factors. Study results showed that skill development was unrelated to gender, age, education level, lifetime work experience, employment tenure, and the number of prior service apprenticeships; other analyses showed the same for the amount of other job-related training the employee volunteers received during the period of the study, whether they managed or supervised others, and the length of time they had managed others. As such, employees' skill development through volunteering appears to be a robust phenomenon that generalizes to employees who differ in numerous ways as just described. Study results did highlight one volunteer characteristic that appeared to matter a great deal for skill development, and those who manage CVPs and who coordinate and manage employee volunteers have opportunity to shape it: self-efficacy about skill improvement.

Study results suggest that employers and nonprofits would be well served by taking steps to increase employee volunteers' confidence in their ability to improve their work-related skills through volunteering. Offering pre-volunteering preparation courses, as Citizen Schools does, coupled with advice for developing goal-oriented strategies that include skill use, and messages about the potential for skill development may be effective ways to bolster self-efficacy. The parties that recruit, coordinate, and manage employee volunteers could emphasize the anecdotal and other evidence for skill development through volunteering, and communicate testimonials from other employees who volunteered in the same or similar contexts. Companies and nonprofits should invest resources to measure and report the effects of volunteering on skill development, and then set goals to promote the strategic design and redesign of volunteer experiences, as other experts have suggested (Points of Light Foundation, 2005). It may also be effective for program coordinators to inform employee volunteers of other potential benefits of skill-based volunteering. For example, Muthuri et al. (2009) found that CVPs through which employees utilized key competencies generated valuable social capital through the building of social networks and relationships with other professionals, Rodell (2013) found that volunteering was linked to higher job performance, and Booth et al. (2009) found that employees who perceived greater skill acquisition reported greater job success and employer recognition.

Increasing self-efficacy about skill development may achieve other ends beyond enhancing employees' professional development. Tomkovick et al. (2008) found that, when university students believed their service-learning projects helped them develop valuable skills, they were more likely to engage in future volunteering. Based on their own findings, Booth et al. (2009) suggested that increasing skill development through volunteering will motivate employees to volunteer more often, and Jones (2010) found that employees who benefited from their employer's CVP reciprocated through extra-role performance related behaviors. In these ways, employee skill development through volunteering may create an ongoing cycle of shared value that provides benefits to employees, their employers, and the communities and causes served by committed employee volunteers.

## Ethics statement

The study design and processes used to protect the interests and rights of the human subjects involved in this study were approved by the Institutional Review Board at The University of Vermont, Committee on Human Research. All subjects provided their informed consent as per the approved protocol for this study.

## Author contributions

The author confirms being the sole contributor of this work and approved it for publication.

## Funding

This research was funded by the Nicole Maria Stata Summer Research Award.

### Conflict of interest statement

The author declares that the research was conducted in the absence of any commercial or financial relationships that could be construed as a potential conflict of interest.
